# CASK and CaMKII function in *Drosophila* memory

**DOI:** 10.3389/fnins.2014.00178

**Published:** 2014-06-25

**Authors:** Bilal R. Malik, James J. L. Hodge

**Affiliations:** School of Physiology and Pharmacology, University of BristolBristol, UK

**Keywords:** CASK, CaMKII, memory, *Drosophila*, mushroom body, calcium imaging, autophosphorylation, disease model

## Abstract

Calcium (Ca^2+^) and Calmodulin (CaM)-dependent serine/threonine kinase II (CaMKII) plays a central role in synaptic plasticity and memory due to its ability to phosphorylate itself and regulate its own kinase activity. Autophosphorylation at threonine 287 (T287) switches CaMKII to a Ca^2+^ independent and constitutively active state replicated by overexpression of a phosphomimetic CaMKII-T287D transgene or blocked by expression of a T287A transgene. A second pair of sites, T306 T307 in the CaM binding region once autophosphorylated, prevents CaM binding and inactivates the kinase during synaptic plasticity and memory, and can be blocked by a TT306/7AA transgene. Recently the synaptic scaffolding molecule called CASK (Ca^2+^/CaM-associated serine kinase) has been shown to control both sets of CaMKII autophosphorylation events during neuronal growth, Ca^2+^ signaling and memory in *Drosophila*. Deletion of either full length CASK or just its CaMK-like and L27 domains removed middle-term memory (MTM) and long-term memory (LTM), with CASK function in the α′/ß′ mushroom body neurons being required for memory. In a similar manner directly changing the levels of CaMKII autophosphorylation (T287D, T287A, or TT306/7AA) in the α′/ß′ neurons also removed MTM and LTM. In the *CASK* null mutant expression of either the *Drosophila* or human *CASK* transgene in the α′/ß′ neurons was found to completely rescue memory, confirming that CASK signaling in α′/β′ neurons is necessary and sufficient for *Drosophila* memory formation and that the neuronal function of CASK is conserved between *Drosophila* and human. Expression of human *CASK* in *Drosophila* also rescued the effect of *CASK* deletion on the activity state of CaMKII, suggesting that human CASK may also regulate CaMKII autophosphorylation. Mutations in human *CASK* have recently been shown to result in intellectual disability and neurological defects suggesting a role in plasticity and learning possibly via regulation of CaMKII autophosphorylation.

## Introduction

**Figure 1:**
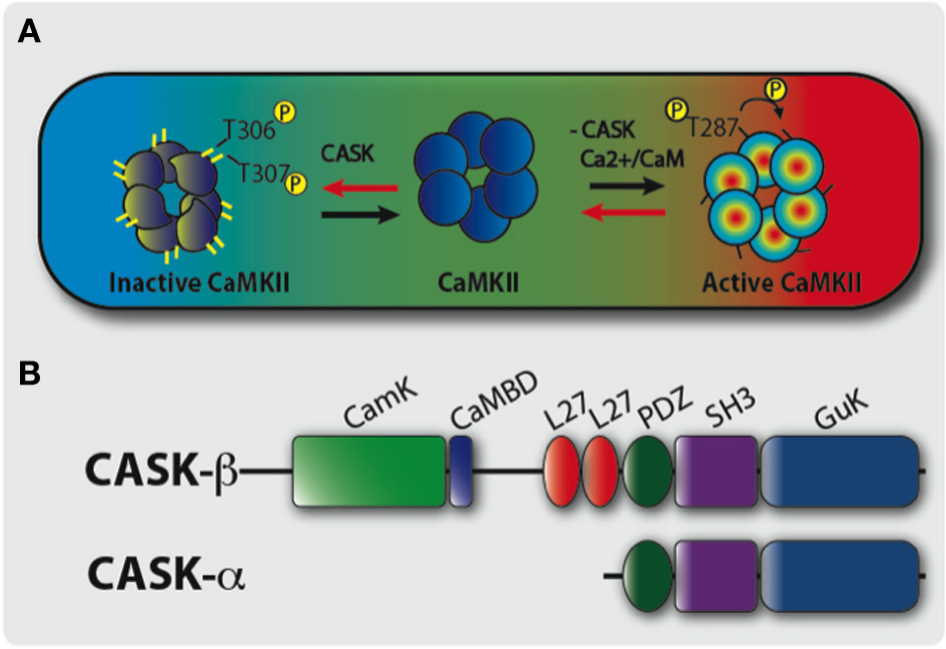
**A model of CASK’s regulation of CaMKII autophosphorylation during memory formation. (A)** The large colored rectangle represents a hypothetical neuron in the middle of which is a cartoon of a single layer of a CaMKII dodecamer holoenzyme. On the right, under conditions of increased synaptic activity (high [Ca^2+^], in red) Ca^2+^/CaM binds CaMKII via the CaM binding site that contains the inhibitory T306 T307 sites hence blocking them from autophosphorylation. This also promotes T287 autophosphorylation (pT287) and the switch to persistently high kinase activity even after Ca^2+^ levels fall. On the left, under conditions of low synaptic activity and low [Ca^2+^] (in blue), there is low probability of CaM binding to CaMKII allowing CASK to promote autophosphorylation of the inhibitory T306 T307 (pT306 pT307) sites. This renders the kinase inactive and even if there is a subsequent increase in Ca^2+^/CaM, CaM binding is blocked by pT306 pT307 in the CaM site. Eventually phosphatases will act to remove phosphorylation events and return endogenous CaMKII to its basal state. Therefore, in the absence of CASK there is a decrease in inhibitory pT306 pT307 and an increase in pT287 constitutively active CaMKII, conversely increased CASK promotes inhibitory pT306 pT307 decreasing pT287 and endogenous CaMKII activity. Therefore, neurons expressing transgenic CaMKII with inhibitory phosphorylation sites mutated to blocking residues (TT306/7AA) or with too little CASK due to mutation results in a form of CaMKII that is unable to switch off. This causes abnormally high CaMKII activity that subsequently interferes with the physiology of the neuron disrupting memory. **(B)** Predicted domain structure of CASK isoforms, the short isoform CASK-α contains PDZ, SH3, and GUK domains while the long isoform CASK-β contains additional CaMK-like (CamK), Calmodulin binding domain (CaMBD), and L27 domains at its N-terminus. The *CASK-β* null contains a N-terminal deletion removing CaMK, CaMBD, and L27 domains but leaves the downstream promoter and whole of *CASK-α* intact (Slawson et al., 2011).

Information is encoded and stored in response to changes in neural activity and Ca^2+^ signaling in circuits underlying memory formation in the brain. One molecule critical for these processes is CaMKII whose activity is acutely sensitive to frequency dependent changes in Ca^2+^ during long-term potentiation (LTP) during hippocampal memory formation (Lisman et al., 2002; Hell, 2014). In addition CaMKII is abundant in structures known to be required for memory and is localized specifically to the parts of the neuron important for memory formation. For instance, CaMKII is the main protein in the hippocampal post-synaptic density (PSD) (Kelly et al., 1984; Hell, 2014) and is similarly enriched in the **mushroom body** memory center of *Drosophila* (Takamatsu et al., 2003; Hodge et al., 2006). CaMKII is thought to act as a molecular memory switch during increased neuronal activity, when increased Ca^2+^ levels stimulate CaMKII autophosphorylation inducing the changes in synaptic strength that underlie learning. This occurs because the increased Ca^2+^/CaM binds to a subunit of the CaMKII dodecamer causing a conformational change exposing a T286 on mammalian CaMKII and T287 on *Drosophila* CaMKII that can be autophosphorylated (Figure 1A), resulting in a Ca^2+^ independent constitutively active kinase (Lisman and Zhabotinsky, 2001). CaMKII knockout mice or treatment with a CaMKII inhibitor resulted in mice with impaired LTP and memory (Silva et al., 1992a,b). Mice expressing either Ca^2+^ dependent CaMKII-T286A or CaMKII-T286D have abnormal LTP and memory (Mayford et al., 1996; Giese et al., 1998; Yasuda and Mayford, 2006). A second pair of autophosphorylation sites (TT305/6 equivalent to *Drosophila* TT306/7, Figure 1A); are exposed in the CaM binding domain when Ca^2+^/CaM dissociates from CaMKII, for instance during low synaptic activity, and are inhibitory as autophosphorylation prevents subsequent CaM binding blocking CaMKII function. CaMKII-TT305/6AA mice show enhanced LTP while CaMKII-TT305/6DD expression also disrupted LTP and memory (Elgersma et al., 2002; Pi et al., 2010). In *Drosophila*, there is no *CaMKII* null, which would be expected to be lethal (Park et al., 2002; Mehren and Griffith, 2004), however peptide inhibition of CaMKII led to synaptic defects and memory deficits in the **courtship-conditioning assay** (Griffith et al., 1993, 1994). Therefore, the control of CaMKII and its autophosphorylation is critical for synaptic plasticity and memory in *Drosophila* and mammals. But the mechanism of regulation of CaMKII autophosphorylation during memory formation is still unclear.

KEY CONCEPT 1 **Mushroom body**

A bilaterally symmetrical neuronal structure in the *Drosophila* brain required for associative memory that is functionally homologous to the mammalian hippocampus. It consists of roughly 2000 neurons that can be subdivided into three classes of intrinsic neurons (α/β, α′/β′, and γ) that extend their axons into five lobes of neuropil.

KEY CONCEPT 2 **Courtship-conditioning assay**

Conditioning is induced by exposure of mature males to previously mated females, who reject his courtship advances. Subsequent exposure of the conditioned males to virgin females, which are normally courted vigorously, results in a suppression of his courtship behavior (e.g., he displays a memory of his previous rejection that can last hours). Although this form of associative learning is more ethologically relevant, olfactory shock conditioning is more widely used as it is easier to apply a variety of CS and US stimuli, for instance appetitive or aversive with the naïve sensory response of the fly to the individual CS and US stimuli being easily controlled for.

One molecule that in addition to CaM regulates CaMKII autophosphorylation is CASK (Figure 1B), a membrane-associated guanylate kinase (MAGUK) synaptic scaffolding protein that contains a CaMK-like and Lin-2/Lin-7 (L27) domain in addition to the canonical PDZ [post-synaptic density protein (PSD95), *Drosophila* disc large tumor suppressor (Dlg1), and Zonula occludens-1 protein (Zo-1)], SH3 (SRC Homology 3), and GUK (guanylate kinase) domains with the CaMK and GUK domains likely kinase dead in *Drosophila* (Hata et al., 1996; Lu et al., 2003). The CaMK domain of mammalian CASK has low levels of Ca^2+^/CaM independent kinase activity against neurexin that unlike other kinases is magnesium independent (Mukherjee et al., 2008; LaConte and Mukherjee, 2013). *Drosophila CASK* has two isoforms, a full-length *CASK-ß* isoform that contains the CaMK-like and L27 domains and PDZ, SH3, and GUK domains (Figure 1B). The other isoform, CASK-α, is short and contains only the common PDZ, SH3, and GUK domains and forms a molecule with structural homology to vertebrate MPP (Slawson et al., 2011). CASK-β associates with CaMKII at synapses and in the absence of Ca^2+^/CaM promotes TT306/7 phosphorylation (Figure 1A), inactivating the kinase (Lu et al., 2003). The function of CASK has also been studied in mice, and while CASK knock-outs are lethal due to a cleft palate phenotype, neurons cultured from these animals show abnormalities in glutamatergic synaptic release (Atasoy et al., 2007). However, the early lethality of these mice prevents the modeling of CASK function in behavior and disease.

Flies completely lacking CASK are viable, have decreased levels of synaptic CaMKII-TT306/7 autophosphorylation and display abnormal habituation behavior in a version of the courtship conditioning assay (Lu et al., 2003). Furthermore, CASK mutants increase T287 autophosphorylation thereby allowing CASK to regulate the CaMKII switch to Ca^2+^ independence (Hodge et al., 2006). CASK is expressed throughout the fly brain including the mushroom bodies (Martin and Ollo, 1996; Lu et al., 2003; Malik et al., 2013). Recently a *CASK-β* mutation that completely removes has been shown to cause a number of cognitive deficits in flies including disrupted sleep and place preference (Slawson et al., 2011; Donelson et al., 2012).

## CASK regulates CaMKII autophosphorylation in the mushroom body α′/β′ neurons during middle-term memory

**Figure 2:**
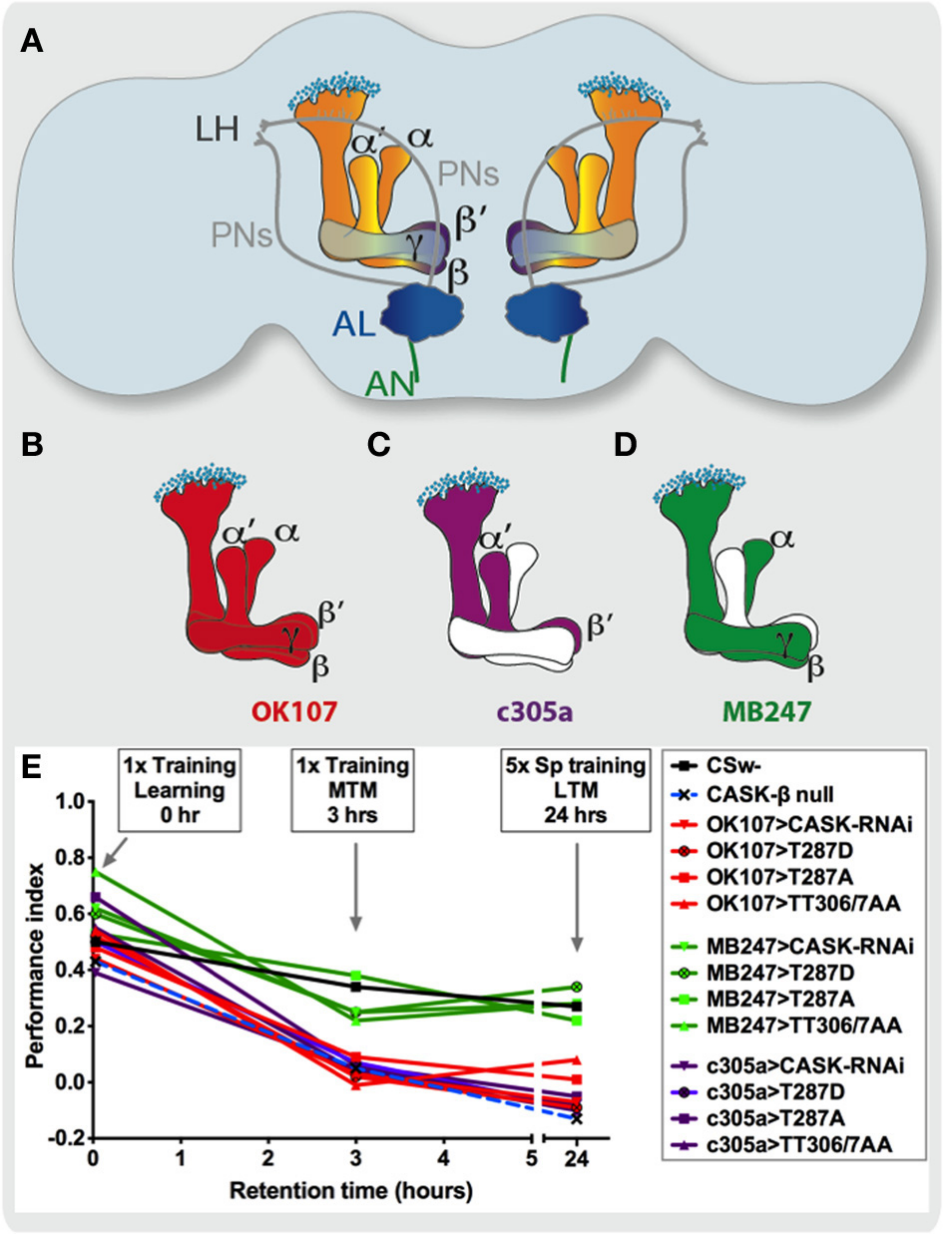
**CASK and CaMKII autophosphorylation function in the mushroom body α′/ß′ neurons during middle and long-term memory formation. (A)** A cartoon representation of a frontal section of the adult *Drosophila* brain showing subdivision of the memory circuit using Gal4 promoters that express in subsets of mushroom body neurons. Olfactory information (CS, conditioned stimulus) is relayed via the antennal nerve (AN) from the olfactory receptor neurons (the first order neurons) to the antennal lobe (AL, dark blue). This information is received in the glomeruli in the antennal lobe which represent the dendrites of the second order neurons, the projection neurons (PNs) send information to the higher brain centers: the mushroom body (the large lobed structures in the center) and the lateral horn (LH) neurons. The mushroom body consists of about 2000 neurons called Kenyon cells whose soma are depicted as small light blue circles. *Drosophila* mushroom bodies consist of three different classes of intrinsic neurons (α/β, α′/β′, and γ) that extend their axons into the five lobes of neuropil that are bilaterally symmetrically arranged in the center of the fly brain (Davis, 2011). **(B)** The *OK107-Gal4* promoter expresses in all mushroom body neurons and a number of neuronal regions outside the mushroom body (in red, Connolly et al., 1996). **(C)** While *c305a-Gal4* (in purple) promoter expresses in the mushroom body α′/β′ neurons as well as other regions (Krashes et al., 2007; Pech et al., 2013) and **(D)** *MB247-Gal4* (in green) expresses in mushroom body α/β and γ neurons (Zars et al., 2000). **(E)** By measuring memory at different times after training, memory retention curves readily depict the effect of each genotype on memory performance **(Performance index)**. Learning or initial (2 min) short-term memory (STM) was measured immediately after administering one cycle of shock-odor training, no statistical difference in learning was seen between CASK and CaMKII genotypes with wildtype (*CSw-* in black). MTM measured 3 h post-one cycle training was completely removed in *CASK-β* null (light blue dashed line) flies. Likewise targeted expression of *CASK-RNAi* (downward triangle), constitutively active *CaMKII-T287D* (black circle with cross in), Ca^2+^ dependent *CaMKII-T287A* (square) and *uas-CaMKII-TT306/7AA* (triangle, both inhibitory sites blocked) throughout the mushroom body (*OK107-Gal4*, red) or just the α′/β′ neurons (*c305a-Gal4*, purple) was sufficient to cause the reduction in MTM and LTM compared to control. Expression of CASK and CaMKII transgenes in the remaining mushroom body α/β and γ neurons (*MB247-Gal4*, green) had little effect. Flies *null* for *CASK-β* or overexpressing *CASK-RNAi*, *CaMKII-T287D*, *CaMKII-T287A*, or *CaMKII-TT306/7AA* throughout their mushroom body or just the α′/β′ neuron completely lacked LTM induced by five cycles of spaced training. Mushroom body α/β and γ neuron expression of *CASK* and *CaMKII* transgenes did not affect LTM.

In order to determine the role of CASK and CaMKII autophosphorylation in learning and memory the *Drosophila* **olfactory shock conditioning** was used (Tully and Quinn, 1985; Malik et al., 2013; Malik and Hodge, in press). All CASK and CaMKII mutant genotypes learned (equivalent to immediate or 2 min memory) similar to wildtype (Figure 2E). When the flies were tested 3 h after training middle-term memory (MTM), the *CASK-β* mutant flies that lack just the long isoform of CASK had reduced MTM. This showed that the CaMK-like and L27 domains only present in this form of CASK (Figure 1B) were the key signaling domains required for memory formation. As *CASK-β* mutations that leave intact PDZ, SH3, and GUK containing *CASK-α*, wiped out memory to a similar extent as a deficiency that removed all forms of CASK (Malik et al., 2013). Previous work has shown that CASK-β regulates CaMKII autophosphorylation by its CaMK-like domain (Lu et al., 2003; Hodge et al., 2006; Gillespie and Hodge, 2013); therefore, it is likely that CASK functions in memory formation via its control of CaMKII autophosphorylation mediated by its CaMK-like domain. We then tested the effect of mushroom body specific reduction of *CASK* on learning (Figure 2A). We used a *UAS-CASK-RNAi* line which reduced the expression of CASK by ~50% (Gillespie and Hodge, 2013; Malik et al., 2013) to test if reduction of CASK in the mushroom body using the ***Gal4/UAS* system** affected memory. Expression of *CASK-RNAi* in either, all mushroom body neurons (*OK107-Gal4*, Figure 2B), or just α′/β′ neurons (*c305a-Gal4*, Figure 2C) similarly showed a drastic reduction in MTM (Figure 2E), while expression in the remaining α/β and γ neurons (*MB247-Gal4*, Figure 2D) had no effect. In order to distinguish the role of CASK in mushroom body development as opposed to an acute physiological role in signaling underlying memory we restricted the reduction of *CASK* to just the adult mushroom body using ***Gal80***^***ts***^ (McGuire et al., 2003; Malik et al., 2013). Reduction of *CASK* specifically in the adult mushroom body α′/β′ as opposed to the adult α/β and γ neurons was sufficient to cause the reduction in MTM showing the effects were post-developmental.

KEY CONCEPT 3 **Olfactory shock conditioning**

Conditioning is induced by the simultaneous presentation of a neutral odor cue (conditioned stimulus, CS^+^) and a reinforcement stimulus, the electric shock (unconditioned stimulus, US); that become associated with one another by the fly. A second conditioned stimulus (CS^−^) is subsequently presented without the US. This is called one-cycle training. During the testing phase, flies are simultaneously presented with CS^+^ and CS^−^ odors in separate arms of a T-maze, and the distribution of the flies in the arms is recorded. To measure long-term memory five cycles of training are given with rests between trails and testing is performed 24 h later.

KEY CONCEPT 4 **Performance index**

The performance index (PI) was calculated as the number of flies avoiding the shock-paired odor minus the flies going to the shock-paired odor, divided by the total number of flies that participated in the test.

Performance index (PI) = (# CS^−^ flies − # CS^+^ flies)/(# total flies)

A score of 1.0 would be equivalent to 100% learning, where all the flies avoided the CS^+^. In contrast a 50:50 distribution would give a PI of zero (no learning).

KEY CONCEPT 5 ***Gal4/UAS* system**

Allows targeted expression of a transgene in any tissue or cell of interest in the fly. Two types of flies are required, the first, the *Gal4* strain contains a copy of the yeast transcription factor (*Gal4*) downstream of a tissue specific promoter sequence. The second fly contains the transgene of interest downstream of an “upstream activator sequence” (*UAS*) that is *Gal4* responsive. By crossing the *Gal4* strain of choice to the *UAS* transgene of interest, one can spatially restrict the overexpression of any gene of interest in the offspring.

KEY CONCEPT 6 **Gal80^ts^**

Addition of the inhibitory *Gal80^temperature sensitive^* transgene allows temporal control of *Gal4* expression by maintaining the flies at 18°C to switch off expression and 30°C to switch on expression.

Decreased levels of CASK are known to increase CaMKII-T287 autophosphorylation (Figure 1A; Lu et al., 2003; Hodge et al., 2006; Gillespie and Hodge, 2013). Consistent with this, we found that direct overexpression of *CaMKII-T287D* in the α′/β′ neurons caused a similar reduction in MTM as knocking-down *CASK* in the same neurons (Figure 2E), while *CaMKII-T287D* expression in the α/β and γ neurons had no effect. Expression of *UAS-CASK-β* just in the α′/β′ neurons of the *CASK-β* null flies fully rescued the MTM defect to a level indistinguishable from wildtype, confirming that CASK signaling in the mushroom body α′/β′ is necessary and sufficient for *Drosophila* MTM formation (Malik et al., 2013).

We also determined for the effect of CaMKII overexpression on memory, showing α′/β′ neuron expression completely removed MTM. In addition reduction of CASK just in neurons that express CaMKII (using a *CaMKII*-specific *Gal4* promoter) was sufficient to remove MTM (Malik et al., 2013). Furthermore, increasing CASK in α′/β′ neurons also greatly reduced MTM. Such increases in CASK would be expected to block T287 autophosphorylation (Hodge et al., 2006), consistent with this idea we found α′/β′ neuron T287A overexpression gave a similar MTM phenotype (Figure 2E). The role of CaMKII-T287 autophosphorylation in the memory neurons is an acute physiological one as opposed to a developmental one, as changing CaMKII-T287 autophosphorylation just in the adult α′/β′ neurons was sufficient to remove memory (Malik et al., 2013). We found α′/β′ neuron overexpression of *CaMKII-TT306/7AA* removed MTM and overexpression of CASK completely rescued the memory deficit due to mushroom body overexpression of *CaMKII-T306/7AA* (Malik et al., 2013). Therefore, our data suggests that CASK regulates CaMKII autophosphorylation during memory in the mushroom body α′/β′ neurons. A role of mushroom body α′/β′ neurons in memory consolidation has previously been proposed (Krashes et al., 2007) however the molecular pathways involved remain largely unknown.

## CASK regulates CaMKII autophosphorylation in the mushroom body α′/β′ neurons during long-term memory formation

Previous work has shown mushroom body overexpression of *CaMKII* or *CaMKII-T287D* enhanced training but did not affect memory in the courtship conditioning assay, while *CaMKII-T287A* overexpression changed habituation and neuronal excitability, but resulted in no change in courtship conditioning memory (Mehren and Griffith, 2004, 2006). However, mushroom body expression of the *CaMKII-RNAi* transgene has been shown to decrease long-term memory (LTM) using the olfactory shock assay (Ashraf et al., 2006) and was associated with decreased mushroom body Ca^2+^ signaling (Akalal et al., 2010). The differences in effects of CaMKII on courtship and olfactory-shock learning phenotypes maybe due to differences in the circuitry employed in the two memory tasks along with the timing of memory measured in the two assays. Recently CaMKII has been shown to undergo CREB-dependent gene transcription and translation in mushroom body and dorsal anterior lateral (DAL) neurons during LTM (Chen et al., 2012). Consistent with these studies we also showed mushroom body expression of *CaMKII-RNAi* only affected LTM (Malik et al., 2013). In addition this is the only *CASK* or *CaMKII* transgene that gave a memory phenotype when expressed in the α/β or γ neuron, suggesting LTM is particularly sensitive and requires a certain baseline level of CaMKII activity in every type of mushroom body neuron in order to form LTM. This is in contrast to α/β or γ neuron expression of CaMKII-T287D, T287A, and TT306/7AA that had no effect on LTM (Figure 2E), possibly because the endogenous CaMKII in α/β or γ neuron maybe sufficient to support enough of the required autophosphorylation activity to generate LTM. This is in contrast to the critical role of α′/β′ neurons that required the correct level of CASK, CaMKII, and CaMKII autophosphorylation in order to form LTM (Figure 2E). Therefore, our data is consistent with the other studies showing α/β or γ neuron expression of *CaMKII-RNAi* disrupts LTM and decreased the peak **GCaMP** Ca^2+^ response, however it should be noted neither study tested a role of α′/β′ neurons (Ashraf et al., 2006; Akalal et al., 2010).

KEY CONCEPT 7 **GCaMP**

GCaMP is a genetically encoded Ca^2+^ indicator, whose expression can be targeted to any neuron of interest using the *Gal4/UAS* system allowing neuronal Ca^2+^ levels to be monitored. GCaMP consists of a fusion of green fluorescent protein (GFP), CaM, and M13; increases in intracellular Ca^2+^ bind CaM causing a conformational change of GCaMP that increases GFP fluorescence on a rapid time scale.

**Figure 3:**
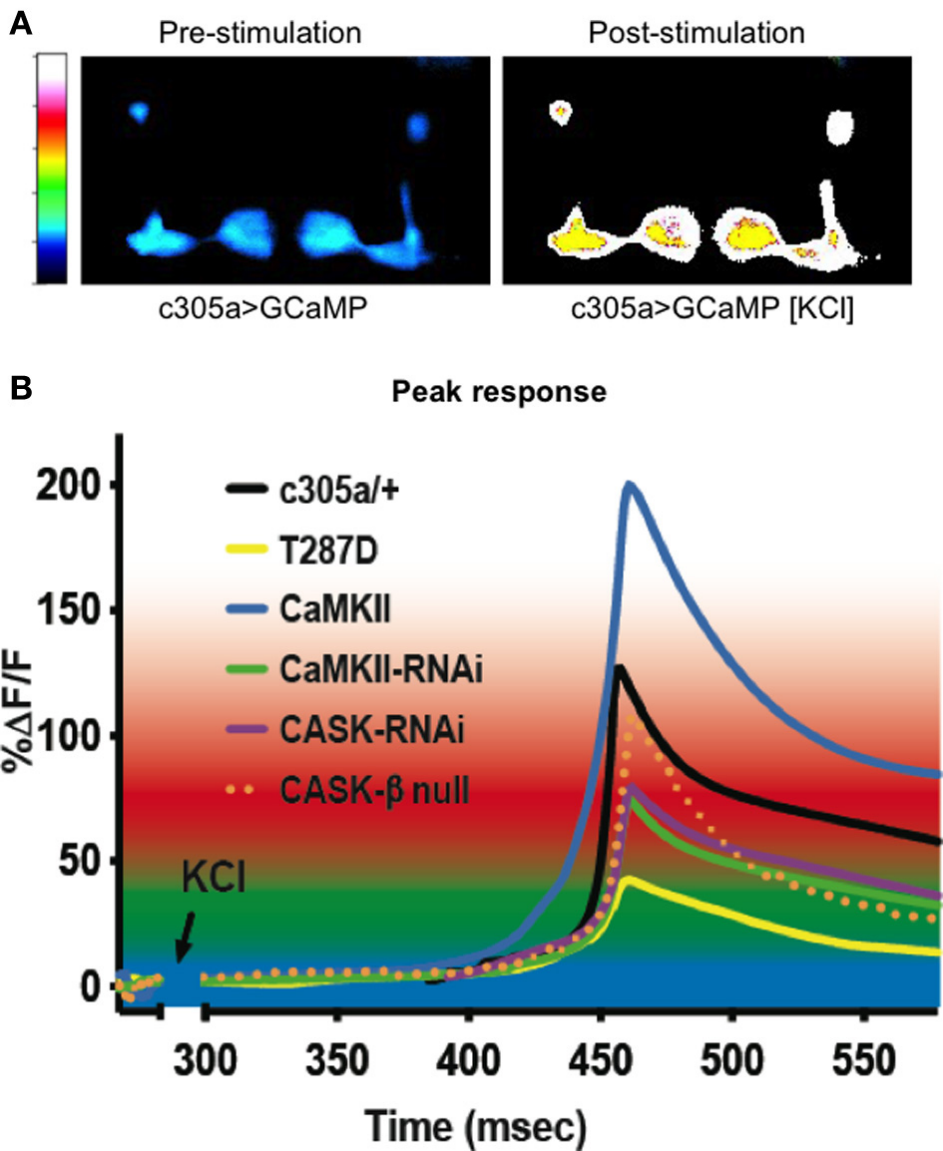
**CASK and CaMKII regulate dynamic changes in neural activity in mushroom body α′/ß′ neurons. (A)** Color coded images of a fly brain showing GCaMP fluorescence in the mushroom body α′/ß′ lobes using *c305a-Gal4* before and after application of depolarizing high [KCl]. **(B)** Traces showing averaged GCaMP fluorescence overtime in the α′/ß′ lobes (*c305a-Gal4*) co-expressing the different CASK and CaMKII transgenes or *CASK-β* null compared to the control c305a/+ expressing GCaMP (solid black line). GCaMP fluorescence is reduced in *CASK-β* null (dotted orange line) and when *CASK-RNAi* (purple line), *CaMKII-RNAi* (green line), or *CaMKII-T287D* (yellow line) were expressed in the α′/ß′ neurons, while *CaMKII* overexpression (blue line) increased the maximum response compared to control.

We also measured a reduction in peak GCaMP Ca^2+^ response in the α′/β′ neurons with *CaMKII-RNAi* (Figure 3); however this was also never tested for in the previous studies. We also found that the reciprocal CaMKII overexpression caused a large increase in peak Ca^2+^ response. Previous electrophysiological studies have shown neuronal expression of *CASK-RNAi* or *CaMKII-T287D* both decreased neural excitability in response to stimulation (Chen and Featherstone, 2011). Likewise we find expression of these transgenes caused a reduction in α′/β′ peak Ca^2+^ signaling. Therefore, the GCaMP data is consistent with the current model of CASK regulation of CaMKII autophosphorylation (Figure 1A; Lu et al., 2003; Hodge et al., 2006) and reveals the likely neurophysiological basis for the disruption of memory resulting from CASK and CaMKII transgene expression in the α′/β′ neurons.

Flies with the *CASK-β* null mutation or reduced CASK in the α′/β′ neurons also reduced LTM (Figure 2E). The LTM effects of CASK could be explained by its role in transcriptional activation of various plasticity molecules including NMDA receptors (Huang and Hsueh, 2009), as NMDA receptors having been shown to be required for *Drosophila* LTM (Wu et al., 2007). We also showed that the CaMKII molecular memory switch (pT287) is required for mushroom body α′/β′ LTM formation with phospho-mimic or block removing LTM (Figure 2E). Again this seems to be an evolutionarily conserved memory mechanism with T286 mutant mice also not being able to form LTM (Irvine et al., 2011).

## Human CASK function in mushroom body α′/β′ neurons restores memory performance of CASK null flies

Point mutations in human CASK have been associated with neurological and cognitive defects particularly involving the cerebellum, including severe learning difficulties resulting from mutations in the CaMK-like and SH3 domains (Najm et al., 2008; Piluso et al., 2009; Tarpey et al., 2009). *Drosophila* and human CASK (74% identical) and CaMKII (79% identical) are remarkably conserved at the protein level, suggesting that they might function in a similar way in both organisms (Cho et al., 1991; Hsueh, 2006). Therefore, we created *UAS-human CASK* flies and expressed the transgene in the α′/β′ neurons of flies that otherwise express no *CASK-β*. Whereas *CASK-β* nulls completely lacked MTM, expression of human *CASK* just in the α′/β′ neurons returned memory to wildtype levels indicating that *Drosophila* and human CASK show conserved neuronal function in memory formation (Malik et al., 2013). We also found that human CASK could regulate *Drosophila* CaMKII autophosphorylation at synapses (Gillespie and Hodge, 2013). Therefore, this work validates the use of *Drosophila* to study CASK and CaMKII in the healthy brain and in disease suggesting a number of directions where this research might lead (Table 1).

**Table 1:** **Outstanding questions and future directions**.

**Animal model**	**Research question**
*Drosophila*	What is the mechanism by which CASK and CaMKII autophosphorylation lead to MTM and LTM formation in the α′/β′ neurons?
	Is the effect pre- or post-synaptic?
	What are the up- and downstream molecules? Do they include EAG potassium channels, NMDA receptors, CREB transcription, and cAMP signaling?
	How do the CaMK-like and L27 domains of CASK-β regulate CaMKII autophosphorylation and memory?
	Does the CaMK-like domain of CASK-β ever show kinase activity? And if so could CASK directly phosphorylate the CaMKII autophosphorylation sites.
	Is this novel pathway ever used in other forms of learning (sugar reward olfactory conditioning or courtship conditioning) or behavior?
Mammals	Does CASK regulate CaMKII autophosphorylation in rodents and human?
	If so, what is the role of this novel form of CaMKII regulation in synaptic plasticity and memory?
	How does CASK mutation lead to brain malformation and mental retardation in human?
	Where does CASK function to regulate memory in mammalian systems?
	Are these effects through abnormal regulation of CaMKII autophosphorylation?
	Does abnormal regulation of CaMKII autophosphorylation contribute to other forms of mental retardation, dementia, or aging?
	Can small molecules or biologicals be developed to treat pathological CaMKII or CASK activity?
	Can *Drosophila* be used to develop models of human CASK and CaMKII diseases allowing screening for these treatments?

### Conflict of interest statement

The authors declare that the research was conducted in the absence of any commercial or financial relationships that could be construed as a potential conflict of interest.
